# The association between diabetes and obesity with Dengue infections

**DOI:** 10.1186/s13098-022-00870-5

**Published:** 2022-07-21

**Authors:** S. D. Sekaran, Z. M. Liew, H. C. Yam, C. S. Raju

**Affiliations:** 1 Faculty of Medicine and Health Sciences, UCSI University Springhill Campus, Port Dickson, 70100 Negri Sembilan Malaysia; 2 Faculty of Applied Science, UCSI University Kuala Lumpur, Kuala Lumpur, 56000 Malaysia; 3 Department of Medical Microbiology, Faculty of Medicine, University Malaya, Kuala Lumpur, 50603 Malaysia

**Keywords:** Diabetes, Obesity, Dengue, Endothelium, Th-1 cytokines, Junctional proteins, Adhesion molecules

## Abstract

Dengue, an arboviral disease is a global threat to public health as the number of Dengue cases increases through the decades and this trend is predicted to continue. Non-communicable diseases such as diabetes and obesity are also on an upward trend. Moreover, past clinical studies have shown comorbidities worsen the clinical manifestation of especially Severe Dengue. However, discussion regarding the underlying mechanisms regarding the association between these comorbidities and dengue are lacking. The hallmark of Severe Dengue is plasma leakage which is due to several factors including presence of pro-inflammatory cytokines and dysregulation of endothelial barrier protein expression. The key factors of diabetes affecting endothelial functions are Th1 skewed responses and junctional-related proteins expression. Additionally, obesity alters the lipid metabolism and immune response causing increased viral replication and inflammation. The similarity between diabetes and obesity individuals is in having chronic inflammation resulting in endothelial dysfunction. This review outlines the roles of diabetes and obesity in severe dengue and gives some insights into the plausible mechanisms of comorbidities in Severe Dengue.

## Background

Dengue fever is caused by Dengue virus (DENV), a Flavivirus from the Flaviviridae family. DENV can be differentiated genetically into 4 serotypes which consists of DENV-1, DENV-2, DENV-3, and DENV-4. The major virus-specific neutralization site is the surface of domain 3 and differs between serotypes [1]. This protein is conserved among the different DENV serotypes but still can be differentiated using monoclonal antibodies. However, some cross-reactivity is sometimes still observed with the different genotypes within the serotypes [2]. In this review, we used databases such as PubMed, Thomson Reuters ISI Web of Knowledge, and Science Direct to look for article pertaining to the following keywords: “Severe Dengue and risk factors”, “Severe Dengue and obesity”, “Severe Dengue and diabetes”, “immune dysfunction in severe dengue”, “chronic inflammation and diabetes”, and “chronic inflammation and obesity”. Using PubMed, keywords such as “Severe Dengue and diabetes’ and “Severe Dengue and obesity” showed only 80 and 26 search results respectively. Only less than 10 search results were found using keyword: Severe Dengue and diabetes and obesity. However, about 95% of the scientific literature used in this review were published between the year 2006 to 2021. This is to ensure that the information provided in this review are relatively new.

Dengue is the most common arboviral disease globally [3]. The number of Dengue cases has increased 4.5 times since the past three decades between 1990 to 2017 [4]. The main vector for Dengue worldwide is *Aedes aegypti* [3] which is commonly found in tropical and subtropical countries causing infections in more than 100 countries [5]. *A. aegypti* originated from sub-Saharan Africa, however, the ancestral form of *A. aegypti* preferred non-human blood [6]. The evolved mosquito also known as the domestic mosquito increased its preference for human blood, possibly due to the alteration of the expression of odorant receptor genes [7]. A change of oviposition of *A. aegypti* is required for survival and adaptation in human habitat which results in oviposition on non-natural environmental areas such as metal, clay or rubber [8]. A wide range of habitat preferences is advantages to *A. aegypti* as this allows for invasion of larger areas at a much faster pace. Kraemer et al*.* (2019) predicted that in the next 30 years 19.96 million km^2^ of land will become available and suitable for *A. aegypti* growth and hence may end up causing 49.13% of the world population to be at risk of Dengue fever taking account climate change, population growth and urbanization [9, 10].

Different dengue serotypes may show different clinical manifestations. According to a cohort study in Singapore, a higher risk for Severe Dengue (SD) is associated with DENV-1 as compared to DENV-2 and DENV-3 based on the criteria published in 2009 by WHO [11]. On the other hand, a retrospective observational study in Malaysia found DENV-2-infected patients have increased risk of developing SD (33%, 9 out of 27 patients) as compared to DENV-1 and DENV-3 [12]. Dengue fever can be classified to 3 different categories according to the WHO 2009 dengue case classification. These are Dengue without warning signs (DWOS), Dengue with warning signs (DWWS) and SD [13]. The clinical presentations of warning signs are abdominal pain, persistent vomiting, clinical fluid accumulation, mucosal bleed, lethargy, liver enlargement, high haematocrit and low platelet count due to blood cell diffusing out from the blood vessels and a decrease in blood volume. Persistence of DWWS may leads to the development of SD with the symptoms of severe plasma leakage, severe bleeding, and severe organ impairment.

The latest version of Dengue classification was suggested with the aim to reduce the misinterpretation of the Dengue severity. Previously according to 1997 WHO Dengue classification guidelines, the Dengue cases were differentiated to three categories, undifferentiated fever, Dengue fever and 4 stages of Dengue haemorrhagic fever (DHF) of which the stages 3 and 4 are defined as Dengue shock syndrome (DSS). The revised classification increased twofold the sensitivity of detecting SD (92.1%) as compared to the 1997 classification (39.0%) [14] and this allowed medical personnel to better manage dengue cases. The hallmark of SD is vascular leakage.

Dengue infection begins with the bite of an infected mosquito which introduces the virus into the body during a blood meal that is required for egg production. The virus is injected directly into the dermis together with salivary fluid, though some researchers feel that the epidermal deposition also occurs which may result in direct inoculation into the blood stream [15]. At the site of inoculation several other factors also are involved such as salivary factors which are thought to reduce macrophage infiltration at the bite site. This may be due to reduced levels of pro-IL-1β and CXCL2 at the bite site [16] and hence inducing innate immune cytokine responses. The salivary protein CLIPA3 of the mosquito spp. *A. aegypti*, has been implicated in facilitating attachment of dengue viral particles to cell surface receptors and in digestion of extracellular matrix [17] for cell migration. During this time several cells get infected which include Langerhans cells, dendritic cells, macrophages, keratinocytes and fibroblasts [18]. This occurs via specific entry receptors such as L-SIGN, DC-SIGN, C-type lectins, the mannose receptor, glycosaminoglycans such as heparin sulphate, TIM-1, TAM, CD14, and CD300a [18] with the DENV E protein structural domain III, which has been implicated as the most likely candidate for binding the cellular entry receptors [19]. As the immune cells circulate in the body, DENV will infect other immune cells and eventually cause viremia. The virus level in the blood reduces when the adaptive immune response is activated with the production of antibodies such as immunoglobin (Ig) M and IgG and the patient eventually recovers from Dengue fever. IgM levels then drop but IgG levels remain high for a period of time. A subsequent dengue infection with a different serotype predisposes to the development of SD with increased inflammation and excessive production of cytokines. Pre-existing IgG antibodies may not have neutralized the virus but instead enables entry into immune cells that bear the Fc receptor. This phenomenon is known as antibody-dependent enhancement. This will result in high titres of DENV and a cytokine storm which eventually leads to SD progression.

T helper cells play an important role in inflammation and the adaptive immune response. There are two major type of T-helper cells which are Th1 and Th2. It is generally known that the type of T helper cells can be differentiated through the identification of the production of cytokines. Th1 cells produce interleukin (IL)-2 and interferon (IFN)-γ [20] while Th2 cells produces IL-4, IL-5, IL-9, IL-10 and IL-13 [21]. Some of these cytokines are also responsible for the polarization of T helper cells and are also involved in creating a balance of Th1 and Th2 responses. Th0, also known as a naïve T cell, is the precursor of T helper cells and has the potential of differentiating into Th1 and Th2 under specific condition during T cell priming. IFN-γ and IL-12 are the key cytokines that determine the cell fate of Th0 to Th1 [22, 23]. Th1 functions as effector cells to activate macrophages through secretion of IFN-γ [24]. These activated macrophages also known as M1 macrophages attract lymphocytes, natural killer cells and neutrophils resulting in pro-inflammatory responses [25]. At the initial Th1 priming stage, IL-12 and IFN-γ are produced by antigen presenting cells [26] and natural killer cells [27] which leads to the initiation of Th1 response. The differentiation of Th1 begins with the activation of signal transducer and activator of transcription factors Stat4 [28] and Stat1 through IL-12 and IFN-γ respectively ultimately induces T-bet expression [29] due to increased accessibility of TBX21 promoter region [30]. As a result, IFN- γ gene is highly expressed and leads to the increased production of IFN- γ [31]. In addition, the activation of T-bet gene and presence of IFN-γ inhibits the expression of IL-4 [23, 32] which prevent the transformation of Th2. For the differentiation of T0 to Th2, activation of Stat5 and Gata3 [33–35] is required and IL-2 appears to be the most potent cytokine to activates this gene compared to IL-4, IL-7, IL-9 and IL-15[36, 37]. IL-4 however activates stat6 which ultimately increases GATA binding protein (Gata 3) expression [38]. This downregulates Stat4 and prevents the Th1 differentiation to occur [39]. IL-4 and IL-10 production from Th2 are well known for their anti-inflammatory properties which mitigates impact of several diseases including autoimmune encephalomyelitis [40] and rheumatoid arthritis [41]. IL4 and IL-13 released from Th2 are also associated with tissue healing and blocking production of Th2-associated protein resulting delayed wound healing in vivo [42]. An imbalance of Th1 and Th2 cytokines may resulting in a poor clinical outcome such as Behcet’s disease [43], Crohn's disease [44] and multiple sclerosis [45] thus the Th1/Th2 ratio should be considered to ameliorate inflammatory diseases.

IFN-γ and TNF-α are Th1 inflammatory cytokines which are responsible for various activities involving the vasculature and endothelial damage. Multiple studies have shown that high levels of IFN-γ and TNF-α are associated with Dengue severity [46–49]. This combination results in endothelial damage in vitro which is mediated through the NO pathway [50]. This was further investigated and showed that addition of NO scavengers and NO synthase inhibitor inhibited endothelial damage caused by these cytokines. On the other hand, another in vitro study showed that IFN-γ promoted apoptosis of endothelial cells possibly due to reduction in the production of nitric oxide [51]. NO is known for its protective role and prevents apoptosis in endothelial cells against various sources including UVA [52], cigarette smoke extract [53], cadmium [54] and ICAM-mediated leukocyte adhesion [55]. On the contrary, however, high levels of nitric oxide may induce apoptosis due to the formation of peroxy-nitrites when reacts with super-oxides [56]. Peroxy-nitrate disrupts the balance of Ca^2+^ in endoplasmic reticulum by increasing the cytosolic Ca^2+^ level and inducing apoptosis possibly through activation of caspases-8 and caspase-9 [57]. Additionally, NO and peroxy-nitrate induces formation of neutrophil extracellular traps (NETs) through phosphoinositide 3-kinases pathway [58]. NETs is composed of neutrophil-released DNA, histones and a variety of proteins [59]. A recent study showed a reduction of NETs by DNase I reduces vascular permeability in a murine model [60]. Citrullinated histone 3, a component of NETs disrupts microvascular endothelial barrier by thinning the adherence junction protein without causing cell injury [61]. Nevertheless, the current studies on the effects of NETs on vascular permeability is relatively vague to draw a conclusive mechanism [62]. Endothelial cells increase expression of VCAM-1 and ICAM-1 when exposed to NETs. A recent clinical study showed high levels of NETs presence among dengue patients which possibly contributes to the progression of dengue pathogenesis [63]. This suggests that NETs may increase vascular permeability in endothelial cells and also induces endothelial activation which eventually increases inflammation causing vascular leakage among Dengue patients.

In diabetes both subsets of T helpers and their respective cytokines have a role to play especially in Type 1 diabetes. Evidence has accumulated indicating a altered Th1/Th2 balance especially in Type 1 diabetes. However different researchers disagree on whether it is Th1 or Th2 mediated [64]. Studies by Sami et al. [65] have shown that both Th1 and Th2 cytokines cooperate in driving b-cell destruction, eventually leading to hyperglycaemia. To date the exact cause is not completely understood. It is established that Type 1 diabetes is associated with a dysregulated immune response, both humoral and cellular and a shift to Th1 favours the pathogenic pathway. Here IL-2 and IFN-γ is said to induce destruction of β-cells by facilitating homing of autoreactive T cells to pancreatic cells and causing their destruction. It is also said that Th2 cytokine particularly 1L-10 accelerated destruction by enhancing the infiltration of these autoreactive cells. However, the mechanics of how these 2 types of helper cells cause the damage is distinct and hence both Th1 and Th2 cells and their respective mediators participate and cooperate in inducing and pancreatic islet β-cell destruction in IDDM [65].

In obesity, the contribution and role of immune cells especially in reference to adipose tissue is still not well established. Adipose tissue is mainly made up of a variety of structural, metabolic and immune cells. These communicate through a network of mediators/cytokines which originate from various immune cells. The presence of the cells and their associated mediators is said to have deleterious consequences on adipose tissue biology. Within the adipose tissue, adipocytes are exposed to the effect of numerous factors, including cytokines, metabolites, hormones and even pollutants, which affect their metabolic capacity and cellular functions. The major cytokines involved are IL-1β which appeared to promote inflammation and metabolic dysfunction in human adipose tissue and are macrophage derived rather than from T cells [66]. In the presence of immune derived cytokines, inflammation is induced which then enhances chemokine production thus attracting more immune cells. The mechanistic actions of Th17 cytokines and IL-1β is still not well understood. Among the other factors produced by the adipose tissues likely candidates include IL-6 and/or TNF-α which are not from T cells but CD45 + cells of the adipose tissue cells. However their kinetics is yet to be determined. With regard to other Th cells another study showed that adipocyte-derived leptin promoted the proliferation and differentiation of CD4^+^ T cells to Th1 cells in mice, and this resulted in increased Th1 cells which induced immune-associated inflammation by producing large amounts of IFN-γ. This promoted adipose tissue macrophages of the M1 phenotype and subsequently caused secretion of proinflammatory cytokines resulting in inflammation and insulin resistance [66].

The biological mechanism of endothelial dysfunction is a common occurrence in certain non-communicable diseases such as diabetes and obesity which may increase the risk of progression to SD disease, probably by affecting the intrinsic permeability of the endothelium of hosts who have been previously infected by another serotype, thus permitting fluid shift to occur. Non-communicable disease burden is also growing and among this group, diabetes mellitus (DM) is emerging with 476 million people worldwide [67]. Observational studies identified DM as an independent risk factor for SD and may be the underlying problem that superimposes the clinical problem due to dengue and hence leading to complications. DM affects the anatomical and physiological integrity of the endothelium as it results in an inflammatory condition due to activation of lymphocytes which leads to the release of pro-inflammatory cytokines. A similar situation is observed in obese children who have a greater risk of infection with dengue viruses and present with more unusual presentations such as encephalopathy and fluid overload [68]. This abnormal lipid environment is associated with vascular complications in diabetes despite current therapies. The Bottom 40% (B40) income group in seeking ways to earn more money, neglect their own health issues like diabetes and obesity and thus increase their risk to SD. In a Brazilian study the risk of dying from dengue is 11-times higher in those with underlying common comorbidities and is much higher with the combination of different comorbidities [69]. In their retrospective analysis comorbidities were identified in a high proportion of dengue deaths and these include renal disease, infectious disease, pulmonary disease and diabetes. Hence individuals with comorbidities require precise access to dengue preventative measures (especially prompt medical care) so as to achieve WHO’s objective of 50% reduction in dengue-related mortality and 25% reduction in morbidity by 2020 [70]. In another study in Pakistan, involving selected comorbidities such as DM, hypertension, bronchial asthma, chronic lung disease, and chronic liver diseases and dengue showed no statistical association with either dengue haemorrhagic fever or dengue shock syndrome [71].

The number of obese and diabetic cases has been increasing to a point that it is now said to be uncontrollable as it becomes a serious public health issue. One of the common things in diabetics and obese individuals are both have chronic inflammation and endothelial dysfunction. It is undeniable that the pathogenesis of SD involving obese and diabetic individuals are linked with each other. This is due to the main symptoms of SD being plasma leakage and involving the vascular endothelium. In this review emphasis is made on the putative roles of these two comorbidities, diabetes, and obesity as risk factors in developing SD more specifically the cytokine profiles and junctional proteins.

## Diabetes: a risk factor for severe dengue

Both dengue and diabetes are epidemic and a large number of individuals in low and middle-income countries are at risk of SD. This may complicate further the clinical presentation of a dengue episode as noted by a systematic review conducted by Htun et al*.* [72] where they proposed that such patients should seek early confirmation and diabetes should be considered in the triage of patients so as to be monitored closely and in a timely manner to avert serious complications and death in patients with acute dengue [72]. In diabetics, it is noted that endothelial dysfunction is a consistent finding. Generally, the repair of the endothelium is accomplished by circulating progenitor cells generally known as endothelial progenitor cells (EPCs) which perform the repairs in physiological and pathological conditions [73]. This is because the endothelium has limited intrinsic capacity of self-repair as it is built up by terminally differentiated cells with a low proliferative potential.

Diabetes generally is associated with chronic inflammation including high level of TNF-α and IL-6 [74–76]. A recent meta-analysis showed that type 2 diabetic patients have high T-helper type 1 cells (Th1)/T-helper type 2 cells (Th2) cytokines including IFN-γ and IL-2 [77]. The cytokine profile of insulin-treated diabetic patients shifts towards Th2 response [78]. A systematic study shows type 2 diabetic patients in Europe and Asia have high nitric oxide levels as compared to healthy individual [79]. Glycation of proteins, lipid peroxidation, malondialdehyde and low antioxidant capacity increases the level of free radicals and inflammation in type 2 diabetes [80]. The devastating effects caused by diabetes needs to be considered and not to be underestimated of its effects towards endothelial damage.

IFN-γ is known to promote internalization of tight junction proteins through endocytosis [81]. This can affect endothelial permeability [82]. IFN-γ also reduces transepithelial resistance of epithelial cells [83]. Occludin, a tight junction protein, colocalizes in the early endosome and this process can be reversed by removing IFN-γ [81]. A further study showed this process to be caused by the activation myosin II which forms the vacuolar apical compartment mediated by Rho-associated kinase pathway [84]. In Dengue TNF-α levels are significantly higher compared to healthy individuals and is elevated in SD [85]. A study in dengue-infected mice showed that anti- TNF antibody is able to reduce dengue-infected mice mortality, with gradual recovery of platelet and erythrocyte counts [86]. The addition of TNF-α enhances ROS and RNS production and apoptosis in dengue-infected endothelial cells [87]. In addition, high TNF-α levels are also associated with decreasing of blood platelet counts [88]. Platelet are needed to promote growth, block gaps and enhance barrier function in endothelial cells [89] while TNF-α is able to reduce Occludin in tight junctions and increase human umbilical vein cells monolayer permeability [90].

The actual mechanism of how tropomyosin affects actin polymerization and conformation is not well known [91]. Previous studies show that the reduction of high molecular weight (HMW) tropomyosin is correlated with poor actin organization [92]. In addition, low levels of HMW tropomyosin resulted in low focal adhesion [93]. Steady-state levels of tropomyosin is required to stabilize actin filament [91]. Actin filaments participate in cell adhesion, focal adhesion, and migration process. At the intracellular level, actin filament can form circumferential actin belt which links it to the adjacent cell and forms the adherence junction. An imbalance of different types of actin disrupts the actin cytoskeleton integrity and causes an increase in permeability in micro vessels [94]. The proteins involved in adherence junction are cadherin and catenin family protein [95] and via phosphorylation of these proteins destabilization of adherence junction occurs within increase endothelial permeability [96]. This eventually will reduce the strength of adherence junction and thus increase vascular permeability.

Tropomyosin 4 levels in plasma is significantly higher in dengue haemorrhagic fever individuals compared to dengue fever individuals [97]. Tropomyosin 1 protein measured using microarray was noted by Soe et al*.* [98] to be highly express in SD patients as compared to Dengue without warning signs [98]. Expression of exogenous brain specific-tropomyosin 1 and 3 reduces focal adhesion but only tropomyosin 3 and brain specific-tropomyosin 3 alter the actin filament arrangement in the cytoplasm [99, 100]. On the other hand, overexpression of TM5NM1 promotes focal adhesion and shows a protective effect against actin sequestering drug [98]. The interaction between tropomodulin 3-Tm5NM1 promotes glucose uptake and insulin-stimulated GLUT4 translocation [101]. Tropomodulin 3 is phosphorylated by Akt which will be bind to Tm5NM1 and this process causes the rearrangement of actin filament which promotes the fusion of GLUT4-storage vesicle leading to increase the number of GLUt-4 on the surface of adipocyte [100]. A study confirmed this finding in a mouse model which showed TM5NM1 promotes glucose clearance and inhibition of this protein caused reduction of GLUT-4 in the plasma membrane of 3T3‐L1 adipocytes [102].

Vascular endothelial (VE)-cadherin is an endothelial specific adhesion molecule located at junctions between endothelial cells. Its main role is in endothelial cell contact integrity and is of vital importance in the maintenance and control of endothelial cell contacts. VE- cadherin also regulates cell proliferation and apoptosis and modulates vascular endothelial growth factor receptor functions. Downregulation and internalization of VE-cadherin disrupts endothelial adhesion junction [103]. Low level of VE-cadherin is said to increase retinal vascular permeability in diabetic rats due to proteolytic degradation this protein and high expression of matrix metalloproteinases (MMPs) [104]. A similar case was observed in diabetic mice model with microvasculopathy where low expression levels of VE-cadherin and Notch 1 were noted [105]. A high glucose environment will increase expression of Orai protein resulting in phosphorylation of VE-cadherin and internalization followed by degradation of VE-cadherin which eventually increases the permeability of aortic endothelial cells [106].

In Type 2 diabetes mellitus (T2DM), atherosclerosis is a key factor that leads to vascular complications. Endothelial dysfunction, hyperglycaemia and excess free fatty acids affects the vascular endothelium by activating a series of events such as platelet hyperactivity, oxidative stress and low-grade inflammation. This enhances vasoconstriction and promotes thrombus formation, ultimately resulting in the development of atherosclerosis. These changes impair the vascular wall and causes cytokine releases and expression of adhesion molecules, platelets get activated and adhere readily to activated endothelium thus mediating leukocyte recruitment, and transmigration which ultimately promotes atherosclerotic vascular complications. Unquestionably, deciphering the of the mechanism of endothelial and platelet dysfunction would enable amelioration of the adverse vascular events that lead to the prothrombotic state in T2DM. In contrast in dengue, platelets are destroyed by an antibody mediated mechanism, rather than by activation but also results in uncontrolled vascular leakage and in some patients in the critical phase develop thrombocytopenia, vascular permeability and plasma leakage. The development of severe disease and poor prognosis are usually associated with pre-existing conditions that affect immune system such as diabetes.

## Obesity: a possible risk factor for severe dengue

Since Malaysia launched World Diabetes Day, many campaigns were carried out to create awareness on the effect of diabetes. The National Diabetes Registry Report indicated that 80.0% and 75.7% of adult diabetes patients in Malaysia had hypertension and dyslipidaemia respectively in 2020 [107]. Malaysia has the greatest number of overweight and obese people in Asia and as many as 7 out of 10 adults suffer from chronic diseases. Obesity-associated insulin resistance is a major risk factor for type 2 diabetes and cardiovascular disease. Many of the endocrine, inflammatory, neural, and cell-intrinsic pathways are shown to be dysregulated in obesity, though it is possible that these factors are interdependent, and a dynamic interplay underlies the pathophysiology of resistance to insulin.

Obesity is often associated with increased risk of many chronic conditions, from diabetes, to dyslipidaemia, to poor mental health and impacts on risk of stroke and cardiovascular disease, certain cancers, and osteoarthritis. The consequences of a global obesity epidemic may also result in a greater global burden of infectious disease owing to obesity and hence infectious disease vigilance is required in populations with high levels of overweight/obesity. Hence a clear need for better clinical practice guidelines for obese individuals is needed. For this, an understanding of these various systems in body will enable proper interventions that specifically prevent or treat insulin resistance and its associated pathologies. These diseases can also affect the patient’s vital organs leading to many complications coupled with the features of metabolic syndrome that may cause damage to the blood vessels. An abnormal lipid environment continues to be associated with devastating vascular complications. It is also said that the intermediary metabolic pathway of de novo lipogenesis is sensitive to insulin [108]. This pathway basically synthesizes lipids from simple precursors, and hence may contribute to these complications. De novo lipogenesis requires fatty acid synthase, and it has been suggested that endogenously produced lipids affect physiology of the endothelium [109].

Obese individuals generally show higher levels of inflammation accompanied by endothelial dysfunction. The number of research articles showing the association between obesity and dengue in recent years have increased. However, the mechanism behind the contribution of these 2 factors (obese or diabetic) in dengue patients, merely addresses a possible association. A meta-analysis in 2018, showed that obesity acts as a risk factor and is correlated with SD among paediatric patients [110]. A retrospective study further showed that obese patients have more devasting clinical manifestations and greater dengue severity [111].

Bandaru et al*.* [112] in his research to link obesity to immune dysfunction, states that obese individuals are more susceptible to various infections. In his studies he showed that leptin alters immune balance in obese individuals and promotes macrophage phagocytosis by increasing secretion of pro-inflammatory cytokines which end up modulating the adaptive immune system [112]. This impairs the immune defence and as a result causes a predisposition to nosocomial, periodontal, respiratory, hepatobiliary, gastrointestinal and postoperative infections. Obese individuals are also said to be hyperleptinemic and this corelates with a decreased antiviral state. Despite this increasing prevalence in both obesity and DENV infections, information linking obesity and dengue directly is still lacking. Another study by Tan et al*.* [111] reported various clinical and laboratory findings in which they showed higher frequency of haemoconcentration, severe thrombocytopenia, elevations of creatinine, liver enzymes, warning sign of increasing haematocrit with rapid drop in platelets and longer duration of hospital stay, which was shown to cause greater disease severity associated with DENV infections amongst obese patients [111]. This further implicates a heightened vigilance that is required when managing this group of patients.

Intercellular adhesion molecules (ICAM)-1 have an important role in regulating the adhesion of the cells and is expressed constitutively at low levels in endothelial cells [113]. High levels of ICAM-1 have been observed in animal models and human subjects. High-fat diet mice have high ICAM-1 levels in their blood plasma, possibly generated from the adipose tissue [114]. Metabolically healthy obese individuals have higher levels of cell adhesion molecules in serum as compared to normal body fat composition individuals [115]. High expression of ICAM-1 can causes disruption of adhesion junctions and endothelial cell barriers and prolong inflammation. ICAM-1 is regulated by MAPK pathway in cerebral and microvascular endothelial cells. Stimulation of ICAM-1 increase the half-life of TNF-α mRNA and induces various inflammatory cytokines [116]. Overexpression of ICAM-1 leads to the loss of endothelial barrier integrity and a disorganized actin cytoskeleton [117]. Interestingly, ICAM-1 activates JNK which leads to the internalization of VE-cadherin and also serves as an inflammatory biomarker as well as retain the transmigration of polymorphonuclear neutrophils [118]. In endothelial cell surfaces of venules, high activity of ICAM-1 may lead to increased solute permeability and absence of leukocytes resulting in low activity of ICAM-1. Without leucocyte-endothelial cell interaction the effects on solute permeability is significantly diminished even in the presence of TNF-α [119]. The expression of ICAM-1 upregulates drastically under proinflammatory condition [120].

Metabolomics studies using quadrupole time-of-flight liquid chromatography tandem mass spectrometry (QTOF-LCMS) revealed that metabolites released in DWOWS, DWWS and SD patients interacted with the lipid metabolism pathway and studies suggested that DENV mediates lipid synthesis and metabolism in their replication cycles [121, 122] by taking advantage of the production of the double membrane vesicles during autophagy for efficient replication [123]. In patients with DWOWS, most of the metabolites are involved in fatty acid metabolism for energy generation and to create triglycerides, phospholipids and other important membrane constituents [124], probably to maintain cellular processes to repair damages upon DENV infection. On the other hand, patients with DWWS and SD expressed large number of metabolites that are involved in the phospholipid metabolism pathway, which regulates the formation and function of the membrane bilayer [122]. Phospholipids are a class of lipids consist of a phosphate group that can form lipid bilayers and function as the major components of cell membrane [125]. Components from different class of phospholipids such as phosphatidylcholine, phosphatidylglycerol and phosphatidylserine as well as phosphatidic acid, which are precursors for other more complex phospholipids were expressed differentially in dengue patients, especially those with DWWS and SD. Deregulated phospholipid metabolism is likely to be due to the changes in exogenous intake of fatty acid from patient’s diet during infection or altered activities of lipid-metabolizing enzymes induced by DENV [126]. Phospholipid metabolism alterations might contribute to membrane permeability destabilization during a DENV infection [127]. Furthermore, metabolites from the sphingolipid metabolism pathway were specifically expressed in SD patients. Sphingolipids are found mainly in the membranes of brain and nervous cells and when altered can result in rearrangement of membrane components that are associated with various neurological diseases [128]. Hence this may be the link to the rare neurological complications, such as brachial neuropathy or encephalopathy that is observed in patients with SD [129].

The pathogenesis of SD is not due to the high viral loads but rather to the hyperactive immune responses that cause loss of homeostasis in inflammation. This may result in uncontrollable immune cell recruitment including leucocytes by the endothelial cells. High levels of macrophage migration inhibitory factor (MIF) is observed in Dengue haemorrhagic patients and has been suggested to increase the expression of thrombomodulin and ICAM-1 [130]. Cytokines that were highly expressed in dengue patients are involved in leukocyte infiltration resulting in proteins in the junctional complex being rearranged and this was noted to be significantly higher in patients with SD. An altered lipid metabolism might be associated with phospholipid metabolism that affects the membrane permeability of microvascular endothelial cells. Apart from this the insulin and cytoskeleton pathway have been also identified to potentially play an important role in the pathogenesis of SD [97]. Hence, regulating the signaling metabolic pathways of lipids, phospholipids and insulin may help towards reducing plasma leakage in patients with SD. Identifying these altered metabolites, proteins and pathways could facilitate dengue diagnosis or be used as a potential target for new therapeutic options.

## Conclusion

It has been suggested that pre-existing immunity to DENV can either protect or exacerbate via antibody dependent enhancement a secondary infection. Worldwide DENV has become a health problem and hence an urgency to understand the mechanisms behind our immune responses both antibodies, T cells, cytokines and a host of other factors that affects this balance between protection versus pathogenesis. However, the mechanisms associating these various factors cells and molecules (both host-related and viral-related factors) is yet to be clearly characterized. The hallmark of severe dengue is vascular leakage and endothelial dysfunction appears as a common biological mechanism by which diabetes and obesity might increase the risk of progression to severe disease, most probably by increasing the intrinsic permeability of the endothelial surface of hosts who have been previously infected by another serotype, thus permitting the occurrence of fluid shift.

Observational studies identified diabetes as an independent risk factor for severe dengue as it also changes the anatomical and physiological integrity of the endothelium due to a permanent inflammatory condition caused by activation of lymphocytes leading to release of pro-inflammatory cytokines. A similar situation is observed in obese children who have a greater risk of infection with dengue viruses and present with more unusual presentations such as encephalopathy and fluid overload. This abnormal lipid environment is associated with vascular complications in diabetes despite current therapies. Endothelial dysfunction may be the common biological mechanism by which diabetes and obesity increase the risk of progression to DHF. The lower income group in seeking ways to earn more money, neglect their own health issues like diabetes and obesity and thus increase their risk to severe dengue. It is also plausible that many diabetics and obese individual experience a first dengue infection that is either sub-clinical or asymptomatic and subsequent infections with different serotypes may exacerbate to greater inflammatory responses on the endothelium. We need to understand the exposures of these individuals to take note of probable increasing incidences of severe dengue in these subsets of cases with underlying comorbidities. These areas require focussed attention especially towards normalizing the aberrant endothelial responses. Lack of a good model of infection as well as access to proper collection of human samples has hampered this progress towards understanding the mechanisms behind the dysfunctional endothelium and more so for vaccine development. To also note that patients in various parts of the world respond differently and this heterogeneity especially requires understanding for therapeutic purposes.

## Data Availability

All the data generated from this study have been provided in the main text.

## Abbreviations

B40: Bottom 40%
DENV: Dengue virus
DHF: Dengue haemorrhagic fever
DM: Diabetes mellitus
DSS: Dengue shock syndrome
DWOS: Dengue without warning sign
DWWS: Dengue with warning sign
EPCs: Endothelial progenitor cells
Gata: GATA binding protein
HMW: High molecular weight
ICAM: Intercellular adhesion molecules
IFN: Interferon
IG: Immunoglobin
IL: Interleukin
MIF: Migration inhibitory factor
MMPs: Matrix Metalloproteinase
NETs: Neutrophil extracellular traps
QYOF-LCMS: Quadrupole time-of-flight liquid chromatography tandem mass spectrometry
SD: Severe Dengue
Th1: T-helper type 1 cells
Th2: T-helper type 2 cells
T2DM: Type 2 diabetes mellitus
VE: Vascular endothelial
